# Effectiveness and Cost-Effectiveness of Using a Social Robot in Residential Care for Individuals With Challenges in Daily Structure and Planning: Protocol for a Multiple-Baseline Single Case Trial and Health Economic Evaluation

**DOI:** 10.2196/67841

**Published:** 2025-08-08

**Authors:** Kirstin N van Dam, Marieke F M Gielissen, Nienke M Siebelink, Ghislaine A P G van Mastrigt, Wouter den Hollander, Brigitte Boon

**Affiliations:** 1 Academy Het Dorp, Research & Advisory on Technology in Long-term Care Arnhem The Netherlands; 2 Tranzo Tilburg School of Social and Behavioral Sciences Tilburg University Tilburg The Netherlands; 3 Care and Public Health Research Institute (Research School CAPHRI) Maastricht University Maastricht The Netherlands; 4 Department of Health Services Research Maastricht University Maastricht The Netherlands; 5 Maastricht HETA Maastricht University Maastricht The Netherlands; 6 Trimbos Institute, Netherlands Institute of Mental Health and Addiction Utrecht The Netherlands; 7 Siza, Center for Long-term Care for People with Disabilities Arnhem The Netherlands

**Keywords:** assistive technology, social robot, executive dysfunction, independence, effectiveness, health economic evaluation, cost of illness

## Abstract

**Background:**

A substantial number of individuals in disability care experience challenges with daily structure and planning and require 24-7 support. The use of a social robot might decrease their need for support from care professionals, leading to improved well-being of individuals with disabilities and increased work engagement for care professionals.

**Objective:**

This paper presents the research protocol for an effectiveness study and a health economic evaluation from a societal perspective on the use of a social robot by individuals experiencing challenges with daily structure and planning who are living in long-term disability care facilities in the Netherlands.

**Methods:**

We will assess the effectiveness of social robot care in reducing the level of support provided by care professionals in a multiple-baseline single case study. In total, 30 participants will be randomly allocated to 1 of 4 clusters, determining the baseline length (2, 3, 4, or 5 weeks) of a 13-week study period, and a 2-week follow-up will be conducted 6 months after participants start using the robot. During baseline, participants will receive care as usual. After baseline, participants will use the robot as part of their care plan. For each participant, 3 to 5 personal goals will be formulated, and attainment of these goals will be evaluated weekly. A health economic evaluation from a societal perspective will be performed to assess the cost-effectiveness.

**Results:**

This study was funded in July 2023. As of October 2024, we enrolled 29 participants. Data collection is planned to be finished in the third quarter of 2025. Data analysis will be performed from the second quarter of 2025. Results will be published in peer-reviewed journals and presented at international conferences in 2026.

**Conclusions:**

We will provide insights into the effectiveness and cost-effectiveness of social robot care for individuals living in Dutch residential care facilities, aimed at enabling them to live more independently, reducing pressure on Dutch care professionals in times of growing staff shortages in long-term care, and allowing care facilities to make informed decisions about implementing such a technology.

**International Registered Report Identifier (IRRID):**

DERR1-10.2196/67841

## Introduction

### Background

In residential disability care, clients (from here on referred to as individuals) rely on care professionals for lifelong support and care. Many of them experience difficulties in facing the complex challenges of daily life, such as managing time, overseeing household tasks, or remembering important information. These challenges are often linked to executive dysfunctions, also known as higher-level cognitive dysfunctions (eg, planning, performing goal-directed tasks, procrastination, attention, taking initiative, and limited time management) [1]. Such dysfunctions are common for individuals with neurodevelopmental disorders (eg, autism spectrum disorder), brain injuries (eg, acquired brain injury), or intellectual disabilities [2,3]. Therefore, these individuals need support in regaining daily structure. In the Netherlands, the size of this group was estimated to be 2 million people in 2024, based on estimates from various publications [4-6]. Approximately 84,000 of them live in a long-term care facility where they receive residential care [7]. Care professionals provide essential support by offering reminders, answering questions, motivating task initiation, giving hints as to where to begin, and engaging in activities together. The individuals are dependent on this external support and may struggle to live independently and participate in everyday life, which in turn impacts their emotional well-being and overall quality of life [8].

Assistive technology holds great potential to offer support to individuals experiencing problems with daily structure and planning in their daily functioning [2]. It may compensate for the gap between a person’s capacity and their everyday life challenges [9], thereby decreasing a person’s dependence on the support of others [10-12]. Moreover, assistive technology offers opportunities to tailor the support to the person’s exact needs, particularly in situations where support provided by care professionals may be limited, insufficient, or unwanted. Systematic review of the literature shows that technology offering functions such as calendars and reminder alarms can improve memory and execution of tasks [13]. However, Brandt et al [13] conclude that further research on the effectiveness and cost-effectiveness of such technologies is warranted. In line with this, the Dutch Ministry of Health, Welfare, and Sport has emphasized the importance of evaluating the impact of innovative technologies in disability care settings to ensure that they are both effective and financially sustainable [14]. Effectiveness studies measure the level of beneficial effect of interventions in “real-world” settings [15,16]. An explorative study [17] found that a social robot that speaks out verbal instructions and reminders at a preset time can support individuals with acquired brain injury, autism spectrum disorder, and mild intellectual disability in remembering and executing daily tasks more independently. This social robot is already widely used in residential care in the Netherlands, particularly in older adult care. In addition, 28 Dutch disability care organizations have reported using this robot in a recent survey of used technology in Dutch long-term disability care [18]. However, both the effectiveness and cost-effectiveness of using a social robot in care are still unknown.

### Aims

We aim to evaluate the effectiveness and cost-effectiveness of using a social robot in Dutch residential disability care for individuals experiencing challenges with daily structure and planning.

We will compare care involving a social robot (CiSR) to care as usual (CAU). The study consists of 2 parts, each with its own focus and research questions (RQs; Textbox 1).

**Research questions (RQs).**

**Effectiveness study**

RQ1: What is the effectiveness of compare care involving a social robot (CiSR) on the support provided by professional caregivers (frequency or duration) compared to care as usual (CAU)?

RQ2: Does the potential effectiveness of CiSR persist at follow-up?

RQ3: Which individual goals are set to be achieved by CiSR, and are these goals attained?

RQ4: What is the effectiveness of CiSR on the well-being of individuals with challenges in daily structure and planning?

RQ5: How does CiSR affect the work engagement of care professionals?

**Economic evaluation**

RQ6: What is the cost-effectiveness of CiSR, assessed by a model-based economic evaluation?

## Methods

This paper presents the research protocol according to the SPIRIT (Standard Protocol Items: Recommendations for Interventional Trials) guideline [19].

### Study Design

A multiple baseline single case study will be conducted in everyday care practice. Multiple baseline single case studies are based on the principle that evidence of a causal effect can be strengthened by assigning participants to baselines of varying lengths. The staggered introduction of the intervention helps to rule out alternative explanations such as maturation or testing and session experience [20].

**Figure 1:**
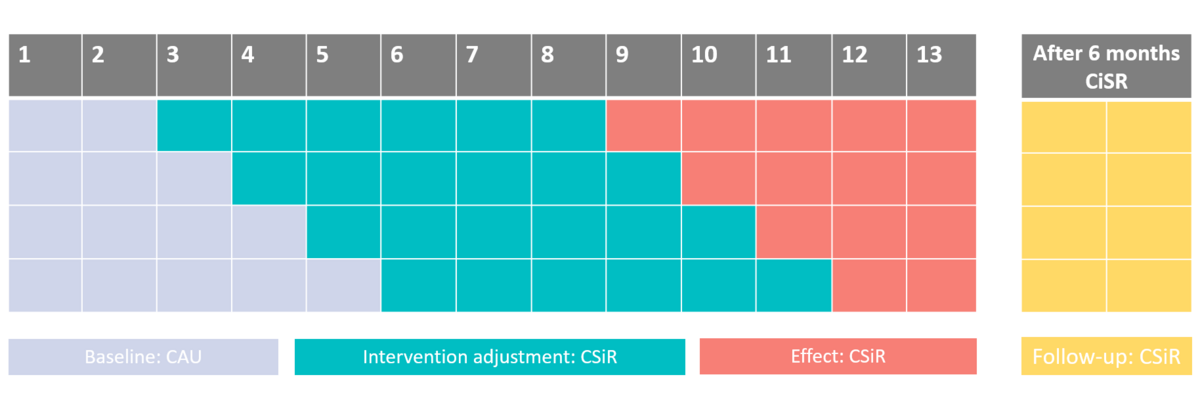
Study planning overview. CAU: care as usual; CiSR: care involving a social robot.

The study period consists of 3 phases (within 13 weeks) and a follow-up phase (Figure 1, Textbox 2, and Table 1). The number and timing of clusters is based on power calculations while considering the practical feasibility. The intervention does not allow for blinding within the trial.

**Summary of the study period.**

- Baseline phase: participants will be randomly allocated to 1 of 4 baseline lengths (2, 3, 4, and 5 weeks, respectively), resulting in 4 different clusters. In the baseline phase, participants will receive care as usual.
- Intervention adjustment phase: a 6-week phase in which each participant starts to receive compare care involving a social robot (CiSR), that is, using and adjusting to the social robot.
- Effect measurement phase: this period varies in length based on the cluster to which the participant is assigned, from 2 to 5 weeks. Participants will continue CiSR.
- Follow-up phase: to observe whether the results are maintained while participants continue CiSR, a follow-up measurement will take place 6 months after the start of the intervention adjustment phase.

**Table 1:** Study planning.

Cluster	Baseline	Intervention	Effect	Follow-up	
1	2 weeks of CAU^a^	6 weeks of CiSR^b^	5 weeks of CiSR	Weeks 34-35	
2	3 weeks of CAU	6 weeks of CiSR	4 weeks of CiSR	Weeks 35-36	
3	4 weeks of CAU	6 weeks of CiSR	3 weeks of CiSR	Weeks 36-37	
4	5 weeks of CAU	6 weeks of CiSR	2 weeks of CiSR	Weeks 37-38	

^a^CAU: care as usual.

^b^CiSR: care involving a social robot.

### Ethical Considerations

This study was approved by the Ethics Review Board Tilburg School of Social and Behavioral Sciences of Tilburg University (TSB_RP1461) and was exempted from ethics approval by the Medical Ethical Review Committee of Radboudumc, Nijmegen (2023-16798). Both the participants and care professionals will be provided with verbal and written information about the study, and their informed consent will be obtained before randomization. To promote full participation in the project, residential care organizations will receive a financial contribution for the research activities, up to a maximum amount of €10,000 (US $10.845,69; currency rate as per October 18, 2024) when different targets are met (such as enrolling 10 participants and completing data collection). Individuals participating in the study will receive no financial compensation. Pseudonymized data will be stored at a secure site with limited access and separated from personal data, such as names. The World Health Organization trial registration dataset is presented in Multimedia Appendix 1.

### Recruitment

#### Participants: Care Organizations

Individuals with challenges in daily structure and planning will be recruited from 3 Dutch residential disability care organizations. Facilities will have to meet the criteria detailed in Textbox 3.

**Inclusion criteria for care organizations.**

- Have an intention to sustainably implement the social robot
- Have an IT infrastructure to support the use of the social robot
- Show commitment to implementation and participation in the research, characterized by the following:
  release funding for the purchase of the social robot
  provide human resources for implementation and training
  provide a project leader to coordinate the project

#### Participants: Individuals With Challenges in Daily Structure and Planning

Individuals will be eligible for inclusion in the study if they meet the predefined inclusion and exclusion criteria, as detailed in Textbox 4.

One coresearcher is recruited from each of the 3 participating care organizations. A coresearcher is an individual who receives care within the context of this care organization and can represent the perspective of the participants. The 3 coresearchers are each coupled with a researcher and will function as a duo. The duos will receive training on how to collaborate and perform coresearch. During the study, coresearch activities will be logged, and the impact of the coresearcher’s involvement on the participants will be evaluated.

**Inclusion and exclusion criteria for individuals with challenges in daily structure and planning.**

**Inclusion criteria**

- Aged ≥18 years (no upper age limit)
- Currently receive residential care
- Struggle with daily structure and planning, for example, have trouble remembering appointments and activities, or require assistance to initiate them
- Capable of understanding and following verbal instructions
- Possess the cognitive capacity to set 3 to 5 personalized goals with the help of a care professional and are motivated to work on these goals with the support of the social robot; these are goals for which the participant usually receives daily or weekly support by a care professional at the start of the study
- Supported by motivated care professionals to guide them in using the social robot

**Exclusion criteria**

- Prone to convert stress into physical aggressiveness
- Overly sensitive to stimuli

### Procedure

#### Overview

The SEAR (Screened, Eligible, Approached, Randomized) framework [21 will be used to systematically monitor and report the recruitment of participnats, according to the CONSORT (Consolidated Standards of Reporting Trials) extension for N-of-1 flowchart [22.

Eligible participants will receive an information letter about the study. The purpose of the research will be explained in a conversation and questions will be answered. The participant and care professional will be given sufficient time to consider the information. When they decide to participate in the study, participants will be asked to sign an informed consent form.

#### Randomization

The researcher (KNvD) will randomly assign each location within the participating care organizations to a cluster. Due to practical considerations, randomization will be applied at the location level rather than the participant level, as this ensures a consistent and manageable implementation for the care team. This cluster determines the duration of the baseline length (2, 3, 4, or 5 weeks). The first 4 locations will be randomly assigned to one of the 4 clusters using a random sequence generator. Subsequent locations will be assigned to the cluster with the fewest participants at that moment. If multiple clusters have the same number of participants, a random sequence generator will be used to decide the assignment. The risk of unequal exposure to external influences is limited due to the initial randomization of the first 4 locations and the balanced allocation strategy of the following locations, which aims to maintain the balance between the clusters.

#### Start of the Study Period

Within each participating care organization, the research period for all locations will begin simultaneously.

### Intervention: CiSR

#### Social Robot (Tessa)

In this study, the social robot Tessa will be used to support the participants. Tessa was developed by Tinybots [23] and was originally designed with and for people with dementia. Tessa is a small wooden egg or flowerpot-shaped robot with amber LED-lit eyes, simulating eye contact and responsiveness. Tessa needs a connection to the power network and the internet via WiFi, MiFi, or cable. The social robot helps support daily structure by providing reminders and suggestions for activities. Tessa does not engage in complex conversations but offers a friendly and predictable presence in the home. From a central location in the user’s home, with a default female voice, Tessa pronounces upcoming appointments or tasks from the user’s day program, with repeated reminders, if necessary. The content of these messages is programmed via a web app, that can be accessed from a smartphone, tablet, or PC, functioning like a simple digital calendar. The app allows users—whether the participant, a care professional, or a family member—to personalize messages, adapting the robot’s communication to the user’s preferences. In this study, the care professional decides on the contents, timing, and frequency of these messages in consultation with the participant. Tessa can either give a reminder or ask a question that can be answered by the user with “yes” or “no.” Depending on the participant’s response, the social robot responds with a preprogrammed message and may give an additional reminder for the task. For example, Tessa asking the user to take a shower in the morning could be programmed with the following message: “Good morning! Did you take a shower?” If the user replies with “yes,” Tessa’s response could be programmed as “Good job, champ!” and if the user answers “no,” Tessa’s reaction could be programmed as “Hop in the shower, and start your day fresh!” The yes or no answers are logged and can be viewed in the web app, enabling the care professional to check task completion without having to ask the client directly.

Each participant will tailor the use of the social robot to their own goals. Before the start of the study, the type, frequency, and duration of support provided by the care professionals, specifically regarding daily structure and planning, will be assessed. In consultation with the care professional, 3 to 5 goals will be selected to work on with the support of the social robot. These can involve (1) activities of daily living (such as brushing teeth, shaving, drinking enough water, and getting up and going to bed on time); (2) household activities (such as doing the dishes, cleaning, changing the bed, and making a shopping list); and (3) planning (such as being ready for an appointment on time or being aware of the daily schedule).

#### Evaluation of Selected Goals by Goal Attainment Scaling Method Once a Week

The selected goals will be formulated using the goal attainment scaling (GAS) method [24]. Setting goals to work on with the support of the social robot is considered an integral part of the intervention and will be evaluated weekly from the start of the intervention adjustment phase. The GAS method is an individualized evaluation method to evaluate the progress of treatment goals, with goals being set according to the SMART (specific, measurable, achievable, relevant, and time-bound) principles and evaluated on a 5-point scale. Therefore, even when the goals of the participants are not similar, the progress on these goals is scored in a similar way. The GAS method is a valid and reliable method [25]. If the participants find it overwhelming to work on all goals simultaneously, a realistic, stepwise schedule will be created.

#### Training of Care Professionals

To integrate the social robot into the care process, care professionals need to be trained through a demonstration and guided practice in using the technology. This will be carried out during a training session, in which an implementation expert will install the social robot together with the care professional or the care team. After this, the care professional will introduce the social robot to the participant. Besides learning how to use the technology, care professionals will need to change their working routines, as the social robot will handle reminders that they previously provided. Thus, they will need to deimplement CAU for these specific goals. This will be discussed with the care professionals during weekly phone calls.

The social robot will be integrated into the care process, with the research team monitoring its implementation by logging and resolving support requests related to Tessa. During weekly phone calls with the care professionals, a researcher will check whether they have encountered any prevailing problems and directly help to solve these problems (eg, technical problems, problems with programming the messages, and communication issues between the various parties involved).

#### Components of CAU

During the baseline phase, the participant will receive CAU from care professionals. The care for individuals with challenges in structure and daily planning usually consists of several components, as described in Textbox 5.

**Components of usual care for individuals with challenges in structure and daily planning.**

- Visual aid: the care professional provides instructions on paper or in digital format
- Hints: the care professional provides verbal instructions before the participant performs an activity
- Supervision: the care professional checks and provides general instructions during or after the performance of an activity
- Verbal instructions: the participant performs an activity while the care professional instructs the participant on what to do
- Demonstration: the care professional shows how to perform the activity, which the participant then replicates
- Doing tasks together: the care professional provides physical assistance or takes over part of the activity, while the participant performs the rest
- Taking over: the care professional performs the entire activity on behalf of the participant, who is not required to be involved.

### Measures

The study uses mixed methods, yielding quantitative as well as qualitative data, to answer the RQs.

#### Effectiveness Study

##### Primary Outcome

The primary outcome variable is the support provided by care professionals to the participant per week. These support moments will be registered daily in a “support diary” for each participant throughout the research period. Each day, the care professionals involved in the support of the participant will log the frequency and duration of each support moment provided to the participant during their shift. On the basis of the intake information provided by the care professionals, the diaries will be standardized and personalized by prefilling the typical daily prevalent types of support relevant to each participant. In this way, the caregivers only need to log frequency and duration of these predefined types of support and provide content descriptions only in cases of nonroutine support. To ensure consistency, the care professionals will receive detailed instructions on how to fill in the diaries by a standardized procedure, which includes participation of the researchers in team meetings and a “test week” before the study period to practice diary logging. Throughout the study, the researcher will hold weekly meetings with a designated care professional at each location to monitor compliance. In addition, to assess interrater reliability of the diary entries, on-site observations will be performed by the researchers.

##### Secondary Outcomes

#### Content of Support Provided by the Care Professionals

The care professionals will log the content of the support they provided to the participants in the support diary. During weekly calls with the care professional, a researcher will also inquire whether there are changes in the content of the provided support and what these changes are.

#### Well-Being of the Participants

The Personal Wellbeing Index–Intellectual Disability will be used to measure well-being. The scale is designed specifically for individuals who have an intellectual disability or other form of cognitive impairment and is considered a state-of-the-art instrument to measure subjective well-being in this population with acceptable psychometric properties [26]. It measures 8 domains of quality of life: life satisfaction, standard of living, health, life achievement, personal relationships, personal safety, community-connectedness, and future security. The Personal Wellbeing Index–Intellectual Disability will be administered 5 times (once every study phase; twice during the intervention adjustment phase).

The 10-item version of the Psychosocial Impact of Assistive Device Scale-Adults measures the psychosocial impact of CiSR, on items such as happiness, independence, self-esteem, and sense of control [27]. The systematic review performed by Atigossou et al [27] supports the use of the 10-item version of the Psychosocial Impact of Assistive Device Scale-Adults and states that it has overall good psychometric properties.

Both questionnaires will be filled in as part of an in-depth interview. During this interview, participants will also be asked about their experiences with the social robot and its added value, with questions such as “What do you find most helpful about Tessa?” or “What would you miss the most if Tessa would be no longer available to you?” The interviews will be guided by a specific topic list, which is available upon request from the corresponding author.

#### Perception of Work by the Care Professionals

To measure work engagement, the care professionals and involved care teams will be asked to complete a short questionnaire, consisting of eight 5-point Likert scale questions, such as “Working with the social robot brings me more enjoyment in my work” or “Working with the social robot helps me save time.”

Complementary in-depth interviews with care professionals will be held to explore their experiences with the implementation of the social robot and how it affects their work engagement. In addition, care professionals will be asked about the perceived added value of CiSR for each client and how it influences the support provided to this participant, with questions such as “How do you perceive the impact of using Tessa on the participant’s progress toward their personal goal?” or “How do you incorporate the use of Tessa into the support you provide to the participant?” The interviews will be guided by a specific topic list, which is available upon request from the corresponding author.

##### Economic Evaluation

###### Medical Consumption and Productivity Cost

Together with the intervention costs, such as the purchase of the social robot and subscription costs, medical consumption and productivity costs will be considered. To measure the medical consumption and productivity costs, a questionnaire containing relevant items from the iMTA Medical Cost Questionnaire and iMTA Assessment Productivity Cost Questionnaire will be conducted at baseline, effect, and follow-up [28,29]. These questionnaires are commonly used to study cost-effectiveness and can be adjusted by selecting relevant items.

###### Quality of Life

To measure the participants’ quality of life, the Icepop Capability Measure for Adults (ICECAP-A) will be administered [30,31]. The use of this questionnaire is advised when performing a cost-effectiveness study [32]. The ICECAP-A contains 5 items measuring stability, attachment, autonomy, achievement, and enjoyment.

Table 2 shows an overview of the measurements.

**Table 2:** Study measurements overview.

Measurement	Baseline	Intervention adjustment	Effect	Follow-up	Used to answer RQ^a^
Daily registration of support (diary)^b^	✓	✓	✓	✓	RQ1 and RQ2
Weekly evaluation of goals (GAS^c^)^b^		✓	✓	✓	RQ3
Interview: well-being (PWI-ID^d^)^b^	✓	✓	✓	✓	RQ4
Interview: psychosocial impact (PIADS-10^e^)^f^			✓	✓	RQ4
Interview: added value of CiSR^f,g^			✓	✓	RQ4
Interview: work engagement and added value of CiSR^h^			✓	✓	RQ3, RQ4, and RQ5
Questionnaire: work engagement^h^			✓	✓	RQ5
Questionnaire: medical consumption and productivity cost (iMCQ^i^+iPCQ^j^)^b^	✓		✓	✓	RQ6 and RQ7
Questionnaire: quality of life (ICECAP-A^k^)^b^	✓		✓	✓	RQ6 and RQ7

^a^RQ: research question.

^b^Information on individuals with challenges in daily structure and planning, provided by the care professional.

^c^GAS: goal attainment scaling.

^d^PWI-ID: Personal Wellbeing Index–Intellectual Disability.

^e^PIADS-10: 10-item Psychosocial Impact of Assistive Devices Scale.

^f^Information on individuals with challenges in daily structure and planning provided by the individuals themselves.

^g^CiSR: care involving a social robot.

^h^Information on care professional.

^i^iMCQ: iMTA Assessment Medical Consumption Questionnaire.

^j^iPCQ: iMTA Assessment Productivity Cost Questionnaire.

^k^ICECAP-A: Icepop Capability Measure for Adults.

##### Analysis

###### Power

Due to the staggered design, power was determined through simulation using the R (R Foundation for Statistical Computing) packages simstudy and lme [33]. For a varying number of participants, 1000 datasets reflecting the staggered design were generated, all assuming a normally distributed outcome variable, a correlation between consecutive weeks of Pearson *r*=0.8, and a final effect of Cohen *d*=0.5 between the baseline phase and the first week of the effect phase (increasing linearly in 6 weeks). With 24 participants, a significant intervention effect (*P*<.05) was observed in 80.3% (803/1000) of cases, between the measurements from the baseline phase and those from the effect phase. Assuming 22% dropout (based on the study by van Dam et al [17]), the study is adequately powered when a total of 24 + (0.22 × 24) = 30 participants are included.

##### Effectiveness Study

Primary outcome data will be analyzed using a multilevel model (R, version 4.0+; package lme4) to investigate whether there is a significant difference between the level of professional care support moments (frequency or duration) per week in the baseline phase and the effect phase. The dependent continuous variable is the frequency and duration of professional care support moments per week. We will analyze all professional care support moments provided, as well as a selection of the specific professional care support moments connected to the goals for which the social robot is deployed.

The independent binary variable is the moment of measurement (baseline phase or effect phase). Because there are multiple measurements per participant at both phases, the model will contain a random intercept for participants. Time will be included in the model as a covariate. Measurements are consequently averaged and compared within participants per phase. Following the intention-to-treat principle, all participants will be included in the analysis, regardless of whether the participant has used the social robot in all weeks during the intervention adjustment phase and effect phase. Because multilevel models handle missing values well, no imputation is needed [34]. Quantitative secondary outcome data will be analyzed similarly. To assess the robustness of the primary outcome, additional per-protocol analyses and completers analyses will be performed as sensitivity analyses.

Clinical relevance will be determined per participant. Here, two conditions must be met as follows: (1) a Reliable Change Index of >1.96 for the level of support provided by care professionals [35] and (2) with CiSR, the participant progresses in carrying out the selected activities independently (GAS score of ≥+1 for at least one goal).

The follow-up data will be used to determine how many participants continue to receive CiSR, whether the effect is sustained in these participants, and what reasons there are to discontinue CiSR.

As recommended by recent single-case intervention research design standards [36], visual analysis will be conducted on the primary outcome. For each participant, data will be presented graphically to compare and interpret changes in patterns, level, and slope within and between different phases (baseline, intervention adjustment, effect, and follow-up). Using the contextual data collected for each case during the study period, we will qualitatively explore and report missing data patterns and discuss their implications for the robustness of the findings.

Qualitative data will be coded and analyzed thematically using Atlas.ti (version 8.4.20; Lumivero, LLC).

##### Health Economic Evaluation

A model-based economic evaluation with a follow-up time equal to the life span of the social robot will be performed. This study will be reported by following the Consolidated Health Economic Evaluation Reporting Standards 2022 guidelines for reporting economic evaluations in health care [37]. This study will also follow the Dutch guideline for health economic evaluations [32].

If the collected data suffices, the cost-effectiveness of CiSR will be examined by a model-based economic evaluation. For this, a Markov model, following the established guidelines [32,38], will be developed. Costs and effects will be modeled for 5 years with cycles of 3 months. The sources for transition opportunities, effects, and cost data will be the effect study, scientific literature, and expert opinions. Cost estimates from the Dutch costing manual will be used as a reference. For costs and health outcomes that occur after 1 year, a 3% discount rate to future costs and a 1.5% discount rate to future health outcomes will be applied [32]. Cost prices will be expressed in euros based on the cost prices in 2024 (index year). If necessary, the existing cost prices will be updated to those in 2024 using the consumer price index available from Statistics Netherlands. Outcomes will be incremental cost-effectiveness ratios using societal costs and effects (the costs per reduced number of support moments), or incremental cost-utility ratios expressed in costs per quality-adjusted life years (QALYs). The QALYs will be calculated for the utilities obtained by the ICECAP-A [39].

For the analyses, R (version 4.0+; package lme4) will be used. Missing measurements will be handled using multiple imputations*.* Resource use and costs will be reported in means, SDs, and 95% CIs. Costs and outcomes will be measured at the baseline (CAU) and the effect and follow-up phases (CiSR). Baseline adjustments will be made if needed. Deterministic and probabilistic sensitivity analyses will be performed. Cost-effectiveness planes will be constructed by plotting the (bootstrapped) cost-effectiveness and cost-utility pairs. The likelihood that CiSR is a cost-effective option compared to CAU will be illustrated using a cost-effectiveness acceptability curve, which demonstrates the probability of an intervention being cost-effective for a given threshold. This represents the amount that society is willing to pay to gain one unit of effect. The willingness-to-pay threshold for the costs per reduced number of support moments is unknown, while the willingness-to-pay for a QALY varies across and within countries. In the Netherlands, the Health Council of the Netherlands estimated the QALY threshold between €20.000 and €80.000 (1=US $1.083; currency rate as per October 18, 2024) depending on the level of severity of the disease [40].

If, in practice, it appears that the data are of insufficient quality, making it impossible to create a model, the costs and effects will be described with a cost-consequence analysis.

## Results

This project received approval for funding on July 13, 2023, by ZonMw, the Netherlands Organization for Health Research and Development (project 10310012210009). The funding period is 30 months.

Data collection from 30 individuals living in residential disability care organizations and the care professionals who provide them support will yield insights into the effectiveness and cost-effectiveness of CiSR. By October 2024, we had enrolled 29 participants. Data collection is planned to be finished in the third quarter of 2025. Data analysis will be performed from the second quarter of 2025. Results will be published in peer-reviewed journals and presented at international conferences in 2026.

## Discussion

### Expected Findings

We expect to demonstrate that the sustainable and person-oriented implementation of a social robot in residential care for individuals with challenges in structure and daily planning will reduce the level of support provided by care professionals. As participants become more independent, the need for support is likely to decrease. This increased independence is anticipated to improve the well-being of participants and the work engagement of care professionals.

In an era of increasing strain on care systems due to rising health care demands and staff shortages, the integration of technology holds substantial promise for improving quality of care. Technology enables more personalized care and might empower individuals to maintain independence in their daily routines, while reducing the need for constant human supervision and support. This may not only improve the quality of life for individuals but also allow care professionals to focus on more complex tasks and meaningful interactions with clients.

Thorough research on the effects, added value, and costs will provide funding bodies and policy makers in long-term care organizations with the necessary information to make informed decisions about implementing technology such as the social robot used in this study. By leveraging these innovations, we can create a more sustainable, effective, and compassionate care system that meets the needs of both individuals and care professionals.

### Strengths and Limitations

We describe the first adequately powered study that investigates the effectiveness and cost-effectiveness of using a social robot in daily residential care practice for individuals experiencing challenges with daily structure and planning. This study will give insight into the characteristics of participants for whom CiSR is successful by combining statistical and visual analysis and considering clinical relevance.

Single-case experimental designs (SCEDs) are increasingly used in behavioral research and provide a clear framework for establishing causal relationships between interventions and outcomes. SCEDs are particularly useful in applied settings and allow researchers to conduct a thorough examination of an individual’s response to an intervention in their daily setting [41]. While enabling analysis of data on a group level due to the multiple baseline design, the design of this study involves frequent measurements for each participant, providing valuable insights into individual effects as well. The use of 4 clusters enhances the internal validity by controlling for potential confounding variables [42]. Given the large variation in support needs regarding daily structure and planning (nature, frequency, and severity) between participants, SCED enables a personalized approach essential to investigating the effectiveness of CiSR [43]. Although personal goals are formulated for each participant, our multiple baseline SCED enables adequate comparison of results between individuals and the application of findings to daily care practice [44]. While the results of group studies may not always apply to individuals, especially in the field of long-term care, SCED focuses on studying how individuals respond to an intervention. This can help understand how to apply the same intervention in the care and support for people with similar conditions [45]. Therefore, with the use of SCED methodology —which allows for an in-depth analysis of individual responses and the identification of causal relationships— findings can be meaningfully interpreted beyond the specific setting [46]. When compared to randomized clinical trials, SCEDs are often questioned for their lack of generalizability. In this study, generalizability is increased by 30 systematic replications (the same intervention, ie, CiSR, with different participants’ characteristics and settings) [47]. This study takes place in a Dutch residential care context. However, given the use of SCED methodology, we anticipate that the findings will be transferable to individuals with similar challenges in residential care settings in other countries.

This study will generate many data points, quantitatively by daily registrations by care professionals, and supported by qualitative data. Here, a potential limitation could be that care professionals might not be able to provide all the expected data due to their heavy workload. This could potentially lead to missing data points. To prevent missing data, we will invest in frequent personal contact with the care professionals involved and take early joint action to offer creative solutions in case of problems arising with the completion of the diaries.

The reason for residential care locations to participate in this study is the desire to sustainably implement and use a social robot. To determine the effectiveness of CiSR, adequate implementation of the social robot in daily care practice by care professionals is essential. The research team will actively stimulate the actual adjustment of work processes to the use of the social robot by investing in weekly contact with the care professionals.

Another strength of this study is the collaboration with coresearchers, which is expected to enable the collection of richer data and a better understanding of the participants and their settings [48].

Finally, this study includes longer-term follow-up assessments, enabling the examination of the sustainable use of the social robot as a part of daily care practice and the maintenance of its potential effects for individuals experiencing challenges with daily structure and planning.

## Appendix

Multimedia Appendix 1 The World Health Organization trial registration dataset.

## Abbreviations

CAU: care as usual
CONSORT: Consolidated Standards of Reporting Trials
CiSR: care involvinv a social robot
GAS: goal attainment scaling
ICECAP-A: Icepop Capability Measure for Adults
QALY: quality-adjusted life year
RQ: research question
SCED: single-case experimental design
SEAR: screened, eligible, approached, randomize
SMART: specific, measurable, achievable, relevant, and time-bound

## Data Availability

The datasets generated or analyzed during this study will not be made publicly available due to the vulnerable position of the participants. However, they will be made available from the corresponding author upon reasonable request.
