# Supplementary material for: Effectiveness and Cost-Effectiveness of Using a Social Robot in Residential Care for Individuals With Challenges in Daily Structure and Planning: Protocol for a Multiple-Baseline Single Case Trial and Health Economic Evaluation
Source: JMIR Res Protoc. 2025 Aug 8;14:e67841. doi: 10.2196/67841 (PMC12374132; doi:10.2196/67841)
Supplement: Multimedia Appendix 1 [file resprot_v14i1e67841_app1.docx]

**Table S1.** The World Health Organization trial registration data set.

| Data category | Information |
| --- | --- |
| Primary registry and trial identifying number | - ClinicalTrials.gov identifier: NCT06592404 |
| Date of registration in primary registry | - September 11, 2024 |
| Secondary identifying numbers | - N/A^a^ |
| Sources of monetary or material support | - ZonMw Programma Goed Gebruik Hulpmiddelen |
| Primary sponsor | - ZonMw Programma Goed Gebruik Hulpmiddelen |
| Secondary sponsors | - N/A^a^ |
| Contact for public queries | - Brigitte Boon, principal researcher - Phone: 0031 88-3779999 - Email: Brigitte.Boon@academyhetdorp.nl - Organization: Academy Het Dorp, Arnhem, the Netherlands |
| Contact for scientific queries | - Marieke Gielissen, coprincipal researcher - Phone: 0031 88-3779999 - Email: Marieke.Gielissen@academyhetdorp.nl - Academy Het Dorp, Arnhem, the Netherlands |
| Public title | - The (Cost)Effectiveness of a Social Robot for Persons with Problems in Daily Structure and Planning in Disability Care |
| Scientific title | - Effectiveness of Using a Social Robot in Residential Care for Individuals With Challenges in Daily Structure and Planning: Protocol of a Multiple Baseline Single Case Trial and Health Economic Evaluations |
| Countries of recruitment | - The Netherlands |
| Health conditions or problems studied | - People with Challenges in Daily Structure and Planning |
| Interventions | - Intervention: social robot (Tessa)  Comparator: regular care (care as usual) |
| Key inclusion and exclusion criteria | - Ages eligible for the study: ≥18 years - Sexes eligible for the study: both - Accepts healthy volunteers: no - Inclusion criteria: adult participant (aged ≥ 18 years), receiving residential care, struggling with daily structure and planning, capable of understanding and following verbal instructions, possessing the cognitive capacity to set and motivated to work on 3-5 personalized goals, supported by motivated care professionals - Exclusion criteria: pica disorder and using a permanent catheter |
| Study type | - Interventional - Allocation: randomized in four clusters - Intervention model: multiple baseline single case design - Masking: none - Primary purpose: care |
| Date of the first enrollment | - February 2024 |
| Target sample size | - 30 |
| Recruitment status | - Recruiting |
| Primary outcome | - Support provided by care professionals per week |
| Key secondary outcomes | - Content of support provided by care professionals - Wellbeing of the person with challenges in daily structure and planning - Quality of life of a person with profound intellectual and multiple disabilities - Perception of work by care professionals - Cost-effectiveness - Cost utility |

^a^N/A: not applicable.
